# An Intriguing Method for Fabricating Arbitrarily Shaped “Matreshka” Hydrogels Using a Self-Healing Template

**DOI:** 10.3390/ma9110864

**Published:** 2016-10-25

**Authors:** Takeshi Sato, Koichiro Uto, Takao Aoyagi, Mitsuhiro Ebara

**Affiliations:** 1 Graduate School of Pure and Applied Science, University of Tsukuba, 1-1-1 Tennodai, Tsukuba, Ibaraki 305-8577, Japan; SATOU.Takeshi@nims.go.jp; 2 International Center for Materials Nanoarchitectonics (WPI-MANA), National Institute for Materials Science (NIMS), 1-1 Namiki, Tsukuba, Ibaraki 305-0044, Japan; flatronl1753s@gmail.com (K.U.); aoyagi.takao@nihon-u.ac.jp (T.A.); 3 Japan Society for the Promotion of Science (JSPS), 5-3-1, Kojimachi, Chiyoda-ku, Tokyo 102-0083, Japan; 4 Graduate School of Industrial Science and Technology, Tokyo University of Science, 6-3-1 Niijuku, Katsushika-ku, Tokyo 125-8585, Japan

**Keywords:** self-healing material, hydrogel fabrication, biocompatible polymer

## Abstract

This work describes an intriguing strategy for the creation of arbitrarily shaped hydrogels utilizing a self-healing template (SHT). A SHT was loaded with a photo-crosslinkable monomer, PEG diacrylate (PEGDA), and then ultraviolet light (UV) crosslinked after first shaping. The SHT template was removed by simple washing with water, leaving behind the hydrogel in the desired physical shape. A hierarchical 3D structure such as “Matreshka” boxes were successfully prepared by simply repeating the “self-healing” and “photo-irradiation” processes. We have also explored the potential of the SHT system for the manipulation of cells.

## 1. Introduction

Polymeric hydrogels are a widely-studied class of biocompatible soft materials that have attracted increasing attention over the last few decades because of their promising applications in broad fields such as food, cosmetics, and biomaterials [1,2,3]. Hydrogels are typically prepared by the polymerization of monomers and cross-linkers in a mold. The resulting gel shape readily depends on the shape of the mold, with relatively simple shapes such as discs, cubes, cylinders, or spheres [4,5,6,7]. However, constructions of complicated, multicomponent self-standing 3D objects with arbitrary shapes have been a big challenge for soft and water-rich hydrogels. New material engineering approaches have to be considered to construct arbitrary shaped gels. In recent years, several sophisticated techniques have been reported for the fabrication of hydrogels with various 3D structures, including photolithography [8,9] and 3D printing [10,11]. Another approach is to attach different blocks or layers after gel fabrication [12,13]. However, these are time consuming and cost-inefficient, and sometimes difficult to produce. Therefore, a stimuli-responsive polymer-based approach has emerged as an alternative method to manufacture complex 3D structures, due to the polymer’s ability change shape in response to external stimuli [14,15]. 

The idea is that a 3D object is obtained by folding, bending, or twisting a programmed 2D structure that consists of stimuli-responsive materials [16,17]. 

Here, we demonstrate the ability of self-healing polymers to act as a template of 3D hydrogel structure with arbitrary shapes (self-healing template; SHT). There has been a growing interest in dynamically-reconstructing or self-healing polymers in recent years because they can undergo automatic healing to repair damage, while stimuli-responsive polymers were designed to function as passive structures. Intrinsic self-healing polymers [18,19], which have a dynamic dissociation and re-association of bonds, are especially an increasingly active research area, because they do not require any curing or adhesive agents. Such dynamic bonds can be accomplished through selective or specific interactions of two complementary compounds, called host–guest interactions [20,21]. Recently, metal–ligand interactions [22] have also gained much attention because they are not only thermodynamically stable, but also kinetically labile. Self-healing hydrogels at room temperature, for example, have been demonstrated using ditopic ligands and lanthanide ions [23]. The combination of catechol–Fe^3+^ bonds have also been known to reconstruct bonds spontaneously [24]. We have recently reported bio-inspired metallo-supramolecular hydrogels using phosphate-terminated poly(ethylene glycol) (PEG-phos) and various trivalent metal ions, such as Fe^3+^, V^3+^, Al^3+^, Ti^3+^, and Ga^3+^ [25]. In this study, we report a new hydrogel fabrication method utilizing our previously developed self-healing hydrogel as a template. 

## 2. Results and Discussion

### 2.1. Preparation of Self-Healing Template (SHT)

**Figure 1:**
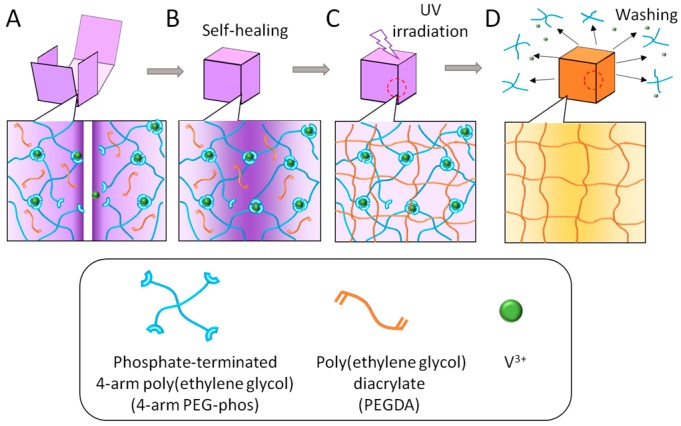
Fabrication processes of 3D hydrogel object using self-healing template (SHT). (**A**) The SHT was first prepared using metal–ligand interactions between trivalent metal ions and four-arm PEG-phos in the presence of PEGDA; (**B**) The SHT was fabricated into a certain shape by folding and bending or assembling each piece; (**C**) The arbitrarily-shaped SHT was then exposed to UV irradiation to crosslink the PEGDA; (**D**) Finally, PEG-phos and metal ions were extracted from the PEGDA gel.

In the present work, arbitrarily shaped hydrogels were fabricated from photo-crosslinkable polymers using the PEG-phos as the SHT. The SHT was first prepared using metal–ligand interactions between trivalent metal ions and four-arm PEG-phos in the presence of a photo-crosslinkable solution, in which PEG-diacrylate (PEGDA) and the photo-initiator irgacure 2959 were added (Figure 1A and Scheme S1). Next, the SHT was fabricated into a certain shape by folding and bending (Figure 1B). The arbitrarily-shaped SHT was then exposed to UV irradiation to crosslink the loaded PEGDA and fix the arbitrary shapes (Figure 1C). Finally, the crosslinked gel samples were transferred into water to remove the SHT from the PEGDA gel (Figure 1D). In the present study, various trivalent metal ions, such as Fe^3+^, V^3+^, Ti^3+^, and Ga^3+^ were examined as crosslinkers. All ions successfully gelated the PEG-phos; however, the gelation time was significantly affected by ion species. For example, gelation quickly occurred within seconds for Fe^3+^, Ti^3+^, and Ga^3+^, while it took 40 s for V^3+^, as previously reported [25]. These phenomena have been explained by their coulomb potential values and water substitution rates [25]. In this study, we have chosen V^3+^ because an optimal period of time is required for shaping and manipulating the hydrogels. In addition, V^3+^ is relatively transparent to UV compared with other metal ions (Figure S1).

### 2.2. Evaluation of Self-Healing Effect on Mechanical Property of Photo-Crosslinked Hydrogels

**Figure 2:**
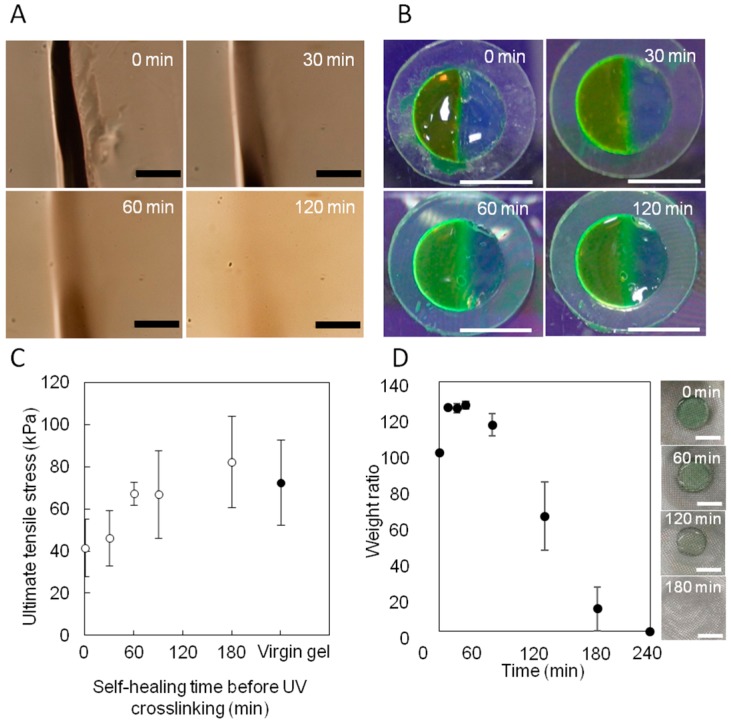
(**A**) Microscopic observation of the fluidic behavior of SHT (scale bar = 200 μm). Time-lapse images show the capability of scratch repair; (**B**) Two SHT samples were prepared, and each sample was cut into two pieces. One of them was colored with Fluorescein isothiocyanate-dextran (FITC-dextran) for clarity. After pressing the fractured surfaces together, they merged into a single piece (scale bar = 1 cm); (**C**) Effect of self-healing time before UV irradiation on the adhesive strength of the resulting PEGDA gels after UV irradiation (*n* = 3, *p* < 0.05); (**D**) Erosion behavior of SHT (scale bar = 1 cm). Mass changes were plotted at the predetermined time (*n* = 3, *p* < 0.05).

Figure 2A shows the dynamic fluidic nature of the PEG-phos SHT at room temperature, as observed by optical microscopy. The scratch gradually disappeared with time, suggesting that the SHT behaves as a liquid-like substrate. However, the SHT did not deform its shape during UV irradiation because of the high viscosity of the SHT. To investigate the time-dependent self-healing process, two pieces of SHT were attached together (Figure 2B). One of them was stained with Fluorescein isothiocyanate-dextran (FITC-dextran; Mw = 3000–5000). After the interfaces came into contact with each other, FITC-dextran started to migrate to the other piece of the SHT. This means that diffusion and reformation of metal–ligand interactions successfully occurred at the interface. To quantify the effect of healing time on the adhesion strength of the joint surface, tensile testing experiments were performed using virgin and healed slab gel samples. The virgin gel samples of SHT containing PEGDA were cut in the middle, and then the two halves were merged together. After standing for 30, 60, 90, and 180 min, they were exposed to UV light for 10 min; 15 mW·cm^−2^ of intensity. The adhesive strength was measured by a loading-to-failure tensile test (Figure 2C). The adhesive strength of PEGDA gels increased with time and reached the same value as virgin gel sample. Thus, it was found that the mechanical strength of a gel was controlled by the diffusion time of the PEGDA. Figure 2D shows the results of an erosion experiment of PEG-phos SHT gel in water. The SHT swelled rapidly, approaching a plateau after 30 min. Then, the SHT started to lose its weight steadily for up to 3 h incubation time. At 4 h, the SHT completely dissolved in water. Although it should take more time for SHT to be extracted from crosslinked PEGDA hydrogels, this result suggested that the metal–ligand interactions between V^3+^-PEG-phos can be easily dissociated by dilution. To determine whether the SHT can be applied for creation of multicomponent hydrogels, two types of SHT gels containing PEGDA with different molecular weights (Mn = 3350 and 10,000) were prepared. The two gels were held in contact with each other and incubated for 180 min and then exposed to UV light for 10 min. The healed hydrogel was immersed in water for 24 h.

### 2.3. Arbitrary Hydrogel Preparation with the Use of SHT

**Figure 3:**
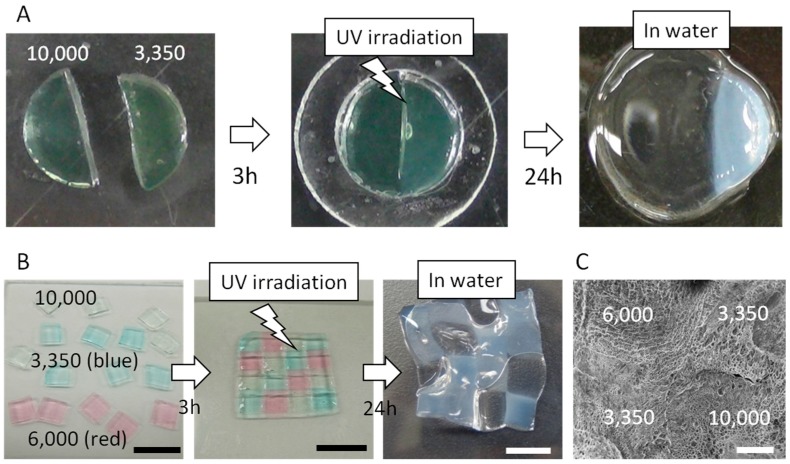
(**A**) Photographs of two SHT samples containing different molecular weights of PEGDA (Mn = 3350 and 10,000). After cutting into two pieces and pressing the fractured surfaces together for 3 h, PEGDA was crosslinked by UV irradiation; (**B**) Photographs of a mosaic-type hydrogel. Three SHT samples containing different molecular weights of PEGDA (Mn = 3350, 6000, and 10,000). After attaching these pieces together, PEGDA was crosslinked by UV irradiation (scale bar = 10 mm); (**C**) SEM image of adhesive interfaces in the mosaic hydrogel (scale bar = 1 mm).

As shown in Figure 3A, a swelling mismatch of the gels was observed. This mismatch induces a large internal stress, because the large swelling region experiences a compression from the less-swelling region. Oppositely, the less-swelling region experiences an extension from the large-swelling region. However, the adhered gels were not broken, and the healed interface did not detach. We have also prepared a mosaic hydrogel using three types of SHT gels containing PEGDA with different molecular weights (Mn = 3350, 6000, and 10,000). The samples with Mn of 3350 and 6000 were stained with blue (methylene blue) and red (methyl red), respectively. The preparation of the mosaic hydrogel was performed in the same way as in Figure 3A. The interfaces of each piece did not detach by the swelling mismatch (Figure 3B). SEM images also confirm there was no fracturing at the adhesive interface (Figure 3C). These results indicate that the SHT system can be utilized to mend two or more different types of hydrogels to create multicomponent objects. In addition to the 2D multicomponent hydrogels, the SHT system can be used to fabricate sophisticated 3D objects.

**Figure 4:**
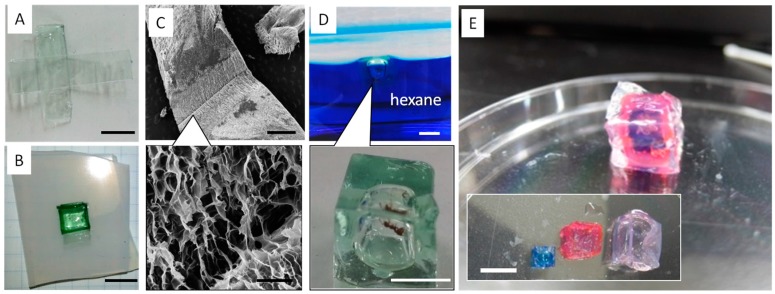
(**A**) A photograph of a 2D planar figure prepared from SHT for a 3D gel cube (scale bar = 1 cm); (**B**) A photograph of a cubic hydrogel box prepared by folding the pre-patterned 2D figure (scale bar = 1 cm); (**C**) SEM images of the adhered interface of the cubic hydrogel box (scale bar = 1 mm and 50 mm); (**D**) Photographs of the cubic hydrogel box floated in hexane (top). An ant was encapsulated in the box. After five minutes, the ant was taken out of the box and was found to be alive (bottom) (scale bar = 1 cm); (**E**) Photographs of the hydrogel boxes before and after nesting (scale bar = 1 cm). Each box was stained with blue (methylene blue) or red (methyl red) for clarity.

We used “paper folding” or “origami” technology to create cubic boxes. First, we prepared the 2D planar figure for a 3D cube using a SHT with PEGDA (Figure 4A). Then, the pre-patterned figure was folded to make a cubic hydrogel (Figure 4B). After the self-healing process to seal the defect areas, the gel was photo-irradiated. SEM images show that the adhesive interfaces were tightly sealed (Figure 4C). To test the air-tightness, the gel box was put into water. The gel box floated in water, and no air or water leakage was observed.

### 2.4. Biomedical Applications of Arbitrarily-Shaped Gels

**Figure 5:**
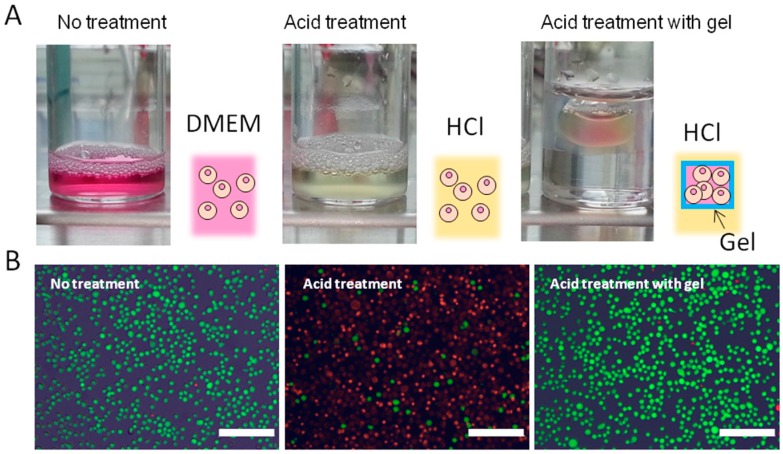

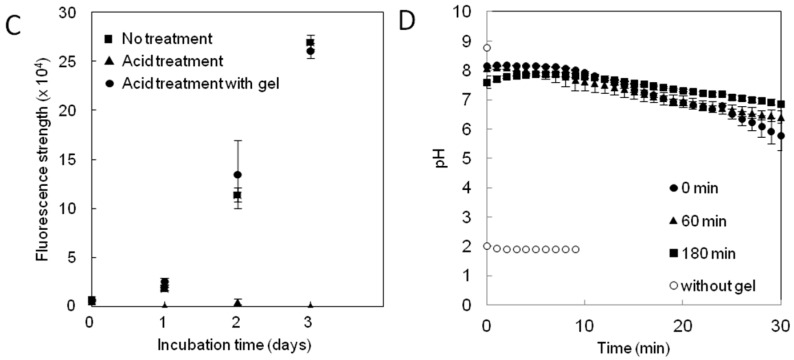
(**A**) Digital photographs of NIH 3T3 cells in Dulbecco’s Modified Eagle’s medium (DMEM), HCl (0.01 M), and in a gel box floating in HCl (0.01 M); (**B**) Live and dead cell assay after each treatment (red; dead, green; alive) (scale bars = 200 μm). The encapsulated cells were collected and reseeded on tissue culture polystyrene (TCPS) dishes; (**C**) Alamar blue assay was used to assess the proliferation of the collected cells after each treatment. Data are presented as mean ± standard deviation (*n* = 2 per condition); (**D**) Time-dependent pH changes of the cell suspended HCl solution without (open) and with (closed) gel box. The gel boxes were self-healed for 0 (circle), 60 (triangle), and 180 (square) min prior to UV crosslink.

To test the applicability of the SHT system, we encapsulated an ant in the box and placed the gel into hexane. After five minutes, the ant was taken out of the box and found to be alive (Figure 4D). We have additionally created hierarchical structures, such as nesting “Matreshka” boxes, by repeating the self-healing and photo-irradiation processes. Figure 4E shows the photographs of the PEGDA hydrogel boxes before and after nesting. Because a facile but tight fixation of the adhered interfaces is considered to be a key point in the creation of hierarchical 3D objects, this SHT system identifies a solution in the field of spatially arranged hydrogel fabrications. Finally, the encapsulated cells using the PEGDA box prepared by the SHT system was demonstrated. Suspension of NIH 3T3 fibroblasts with a density of 1.0 × 10^6^ cells mL^−1^ was placed in the box. The box was then immersed in acidic solution (HCl 0.01 M) for 10 min (Figure 5A). The encapsulated cells were collected and reseeded on tissue culture polystyrene (TCPS) dishes. A live/dead assay of the collected cells showed that almost all the cells were alive, whereas very few cells were alive when they were directly injected into the HCl solution without the gel box (Figure 5B and Figure S2).

An alamar blue (AB) assay was also performed to determine the proliferation of the collected cells. The collected cells were reseeded on TCPS dishes, and proliferation was observed. The proliferation profile did not differ from that of control cells that were directly injected into cell culture medium (Dulbecco’s Modified Eagle’s medium, DMEM) without the gel box (Figure 5C). As expected, HCl treated cells without the gel box did not proliferate due to the acute toxicity of the acidic conditions. Figure 5D shows time-dependent changes in pH within the gel box when it was immersed in HCl solution. Although the pH gradually decreased due to the diffusion of proton ions through the gel, the pH value was still maintained for 10 min.

## 3. Methods

### 3.1. Materials

Four-arm poly(ethylene glycol) (4-arm PEG) (Mn = 40,000) and linear PEG (Mn = 6000) were provided by NOF Co., Ltd. (Tokyo, Japan) and purified by precipitations in hexane. 2-Hydroxy-4′-(2-hydroxyehoxy)-2-methylpropiophenone (irgacure 2959) and linear PEG (Mn = 3350 and 10,000) were purchased from Sigma-Aldrich Co., LLC. (St. Louis, MO, USA). Tetrahydrofuran (THF) ultradehydrated, diisopropylamine, titanium(III) chloride solution (20%), iron(III) chloride hexahydrate, and phosphoryl chloride were purchased from Wako Pure Chemical Industries Ltd. (Osaka, Japan) and used as received. Acryloyl chloride was purchased from Tokyo Chemical Industry Co., Ltd. (Tokyo, Japan) and used as received. Vanadium(III) chloride hexahydrate was purchased from Thermo Fisher Scientific Chemicals Inc. (Waltham, MA, USA) and used as received.

### 3.2. Polymer Synthesis

The preparation of terminal phosphorylated four-arm PEG (4-arm PEG-phos) was carried out as follows. Four-arm PEG (Mn = 40,000) with hydroxyl end group was dissolved in 300 mL of THF (1.67% *w*/*v*). POCl_3_ was dissolved in 200 mL of super dehydrated THF (5% *v*/*v*), and the solution was kept at ca. 0 °C with an iced bath. The PEG solution was then added into the POCl_3_ solution. Diisopropyl amine was also added to the PEG and POCl_3_ mixture to remove the generated HCl. The mixture was then stirred at room temperature for 24 h. After the reaction, THF was totally evaporated using a rotary evaporator, and the residue was dissolved in 200 mL of water. This aqueous solution was then dialyzed for 3 days against water using a dialysis membrane (molecular weight cut off = 3500). The dialyzed aqueous solution was then lyophilized to obtain phosphate-terminated PEG as a white powder.

### 3.3. Preparation of Poly(ethylene glycol) Diacrylate (PEGDA)

Linear poly(ethylene glycol) (Mn = 3350, 6000, or 10,000, 1 eq. mole amount) was dissolved in 50 mL of THF. Acryloyl chloride of 132.5 eq. molar relative to the terminal group of poly(ethylene glycol) was diluted with dichloromethane. The acryloyl chloride was slowly added into the PEG solution by cooling with an ice bath. The reaction solution was gently bubbled by N_2_ gas overnight to remove HCl from the solution. After the reaction, the excess amount of acryloyl chloride was removed by precipitating into diethyl ether three times. A white precipitate was collected by vacuum filtration and then dried in vacuo.

### 3.4. Hydrogel Preparation Using Self-Healing Templates (SHTs)

The 4-arm PEG-phos and PEGDA were dissolved in an irgacure 2959 (10 wt %) solution at concentrations of 41.3 g/L and 125 g/L, respectively. A 0.3 M vanadium chloride aqueous solution was prepared, and PEGs solution (800 μL) and vanadium chloride solution (50 μL) were mixed together. The mixed solution was immediately poured into a mold that was made from a glass slide (50 mm × 50 mm) with silicone rubber of 1 mm thickness. The solution was then incubated for a few minutes to cross-link the self-healing hydrogel network.

### 3.5. Optical Analysis of Metal Ion Solutions

Aqueous solutions of titanium(III) chloride, iron(III) chloride hexahydrate, and vanadium(III) chloride hexahydrate at a concentration of 8.3 × 10^−3^ M were prepared. Absorbance of these solutions was characterized from 300 to 600 nm using a Jasco V-650 spectrophotometer (Jasco Co., Tokyo, Japan).

### 3.6. Tensile Tests

Dumbbell-shaped SHTs were cut in half using a razor blade. Two pieces were pushed together so that their surfaces came into contact with each other. After standing for a predetermined time (0, 30, 60, 90, and 180 min), the samples were exposed to UV irradiation (using Optical Modulex SX-U1251HQ, Ushio, Tokyo, Japan) to cross-link the PEGDA gel. The gel was immersed in water for 1 day to dissolve the self-healing network. The gel samples were subjected to tensile tests utilizing a tensile testing machine (EZ-S 500N, Shimadzu, Kyoto, Japan). These samples underwent tensile tests at 3 mm/min until the gel specimens fractured. 

### 3.7. Preparation of Arbitrarily-Shaped Hydrogels

Prepared SHTs were cut, folded, and then attached to form a predetermined shape. The interfaces underwent the self-healing process for an appropriate amount of time, typically 60 min. The self-healed SHTs were exposed to UV light (15 mW·cm^−2^) for 10 min in order to form photo cross-linked hydrogel networks. Photo cross-linked networks were then immersed in water for at least one day to remove V^3+^ ions.

### 3.8. Cell Culture

Suspension of NIH 3T3 fibroblasts with a density of 1.0 × 10^6^ cells mL^−1^ were put in a box (1 cm^3^)-shaped gel with an injection pump, and then the gel was placed in 4 mL of HCl (0.01 M) for 10 min. The encapsulated cells were collected and reseeded on tissue culture polystyrene (TCPS) dishes in Dulbecco’s Modified Eagle’s medium (DMEM) in the presence of 10% fetal bovine serum (FBS) at 37 °C for 72 h. A live/dead assay was performed to determine the number of viable and non-viable cells. The collected cells were treated with 500 μL of 2 μM calcein AM (positive) and 4 μM EthD-1 (negative) solution for 30 min at room temperature, and then observed by fluorescence microscopy (IX71, Olympus, Tokyo, Japan). Calcein AM and EthD-1 produced green and red fluorescence at 488 nm and 543 nm, respectively. For the evaluation of cell viability, the cells underwent an alamar blue (AB) assay after 3 h, 24 h, 48 h, and 72 h. As negative and positive control, the same type of cells were treated by 4 mL of 0.01 M HCl for 10 min and DMEM containing 10% FBS, respectively. The collected cells were treated with an AB solution. The supernatants were placed into a 96-well micro plate, and the absorbance was measured at 590 nm using a micro-plate reader (Bio-Rad Laboratories).

## 4. Conclusions

A self-healing template-based approach was successfully demonstrated for the creation of arbitrarily-shaped hydrogels. By using this technique, multicomponent 2D gels were successfully prepared. In addition, it has also been applied to fabricate sophisticated 3D objects, such as “Matreshka” boxes. The prepared hydrogels showed tight sealing of the adhesive interfaces without the use of sutures. This approach will provide a robust and facile method for the manipulation and delivery of living cells as well as the formation of tissues mimicking native tissue constructs.

## Supplementary Materials

The following are available online at www.mdpi.com/1996-1944/9/11/864/s1. Scheme S1 shows schematic illustration for synthesis of 4-arm PEG-phos and PEGDA. Figure S1 indicates the result of absorbance spectra of FeCl_3_, TiCl_3_ and VCl_3_ aqueous solution (8.3 × 10^−3^ M each). FeCl_3_ solution shows the absorbance wavelength of 300–400 nm. This wavelength range is utilized for UV crosslinking of PEGDA gel. Figure S2 shows the result of Cell viabilities obtained from live/dead assays.
