# Supplementary material for: An Intriguing Method for Fabricating Arbitrarily Shaped “Matreshka” Hydrogels Using a Self-Healing Template
Source: Materials (Basel). 2016 Oct 25;9(11):864. doi: 10.3390/ma9110864 (PMC5457250; doi:10.3390/ma9110864)
Supplement: Supplementary file 1 [file materials-09-00864-s001.pdf]

Supplementary Materials: An Intriguing Method for Fabricating Arbitrarily Shaped "Matreshka" Hydrogels Using a Self-Healing Template

Takeshi Sato, Koichiro Uto, Takao Aoyagi and Mitsuhiro Ebara

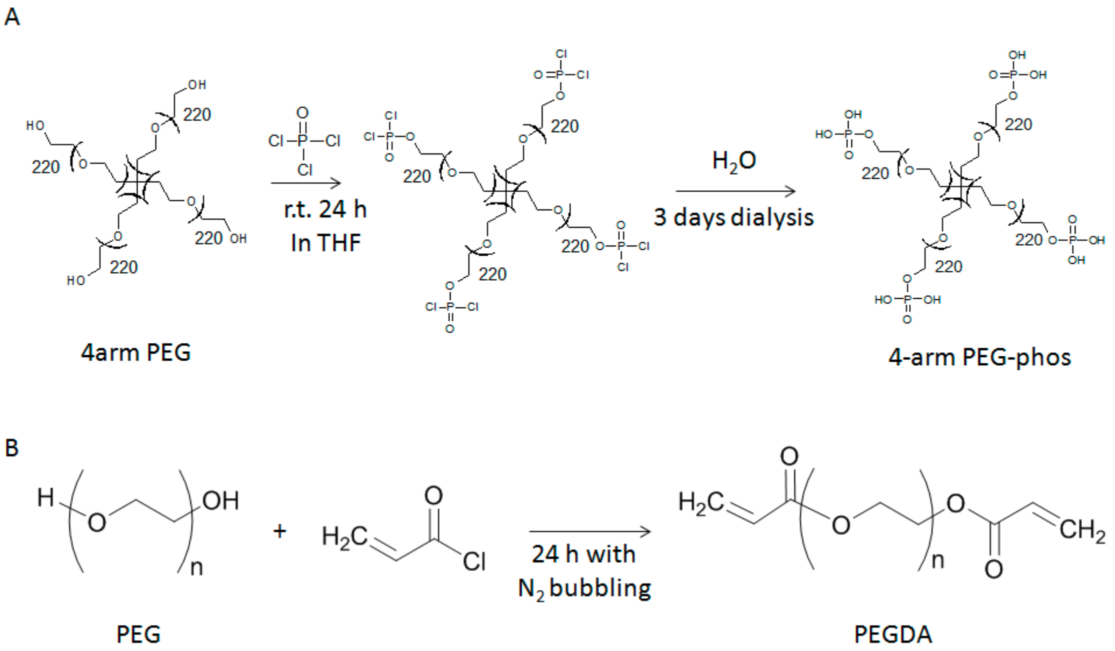

Scheme S1. Synthesis of 4-arm PEG-phos (**A**) and PEGDA (**B**).

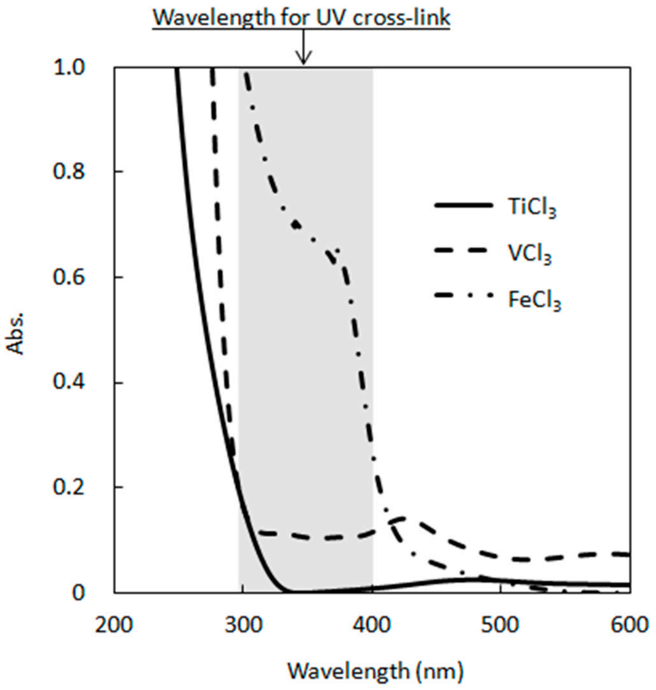

Figure S1. Absorbance spectra of $FeCl_3$, $TiCl_3$ and VCl_3 aqueous solution (8.3×10^{-3} M each). $FeCl_3$ solution shows the absorbance wavelength of 300–400 nm. This wavelength range is utilized for UV crosslinking of PEGDA gel [1,2].

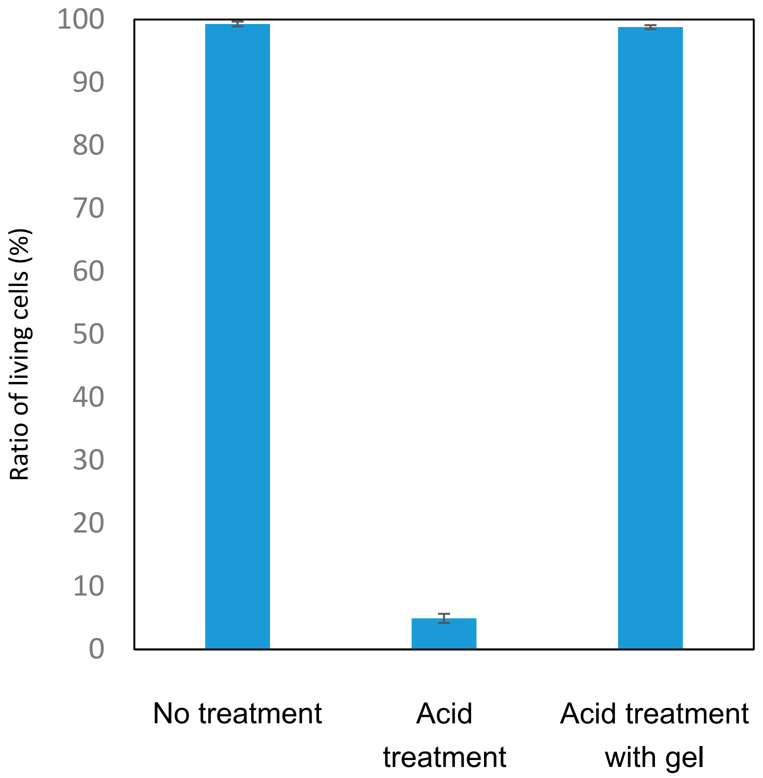

Figure S2. Cell viabilities obtained from live/dead assays.

References

1. Yang, J.; Vitale, A.; Bongiovanni, R.; Nie, J. Synthesis and characterization of siloxane photopolymers used for microfluidic devices. *New J. Chem.* **2015**, *39*, 2532–2540.
2. Calldorera-Moore, M.; Kang, M.K.; Moore, Z.; Singh, V.; Sreenivasan, S.V.; Shi, L.; Huang, R.; Roy, K. Swelling behavior of nanoscale, shape- and size-specific, hydrogel particles fabricated using imprint lithography. *Soft Matter* **2011**, *7*, 2879–2887.
